# Exploring the Behavior of Bovine Serum Albumin in Response to Changes in the Chemical Composition of Responsive Polymers: Experimental and Simulation Studies

**DOI:** 10.3390/polym8060238

**Published:** 2016-06-18

**Authors:** Shih-Rong Hsieh, P. Madhusudhana Reddy, Chi-Jung Chang, Awanish Kumar, Wan-Chi Wu, Hui-Yi Lin

**Affiliations:** 1 Department of Surgery, Taichung Veterans General Hospital, 1650 Taiwan Boulevard Section 4, Taichung 40705, Taiwan; Season@vghtc.gov.tw; 2 Department of Chemical Engineering, Feng Chia University, 100, Wenhwa Road, Seatwen, Taichung 40724, Taiwan; pmsreddy86@gmail.com (P.M.R.); amypc82@gmail.com (W.-C.W.); 3 Department of Chemistry, Massachusetts Institute of Technology, Cambridge, MA 02139, USA; awanishmphil@gmail.com; 4 School of Pharmacy, China Medical University, 91, Hsueh-Shih Road, Taichung 40402, Taiwan; hylin@mail.cmu.edu.tw

**Keywords:** protein, responsive polymer, thermal stability, folding, unfolding, biomedical applications

## Abstract

Knowledge of the interactions between polymer and protein is very important to fabricate the potential materials for many bio-related applications. In this regard, the present work investigated the effect of copolymers on the conformation and thermal stability of bovine serum albumin (BSA) with the aid of biophysical techniques such as fluorescence spectroscopy, circular dichroism (CD) spectroscopy and differential scanning calorimetry (DSC). In comparison with that of copolymer PGA-1.5, our fluorescence spectroscopy results reveal that the copolymer PGA-1, which has a lower PEGMA/AA ratio, shows greater influence on the conformation of BSA. Copolymers induced unfolding of the polypeptide chain of BSA, which was confirmed from the loss in the negative ellipticity of CD spectra. DSC results showed that the addition of PGA-1 and PGA-1.5 (0.05% (*w*/*v*) decreased the transition temperature by 14.8 and 11.5 °C, respectively). The results from the present study on the behavior of protein in response to changes in the chemical composition of synthetic polymers are significant for various biological applications such as enzyme immobilization, protein separations, sensor development and stimuli-responsive systems.

## 1. Introduction

Synthetic polymers, particularly stimuli responsive polymers, have received extensive attention and opened a broad field of potential applications in bioimaging, drug delivery, tissue engineering and bioactive surfaces [1,2,3,4,5], due to their extraordinary response to changes in temperature, pH, ionic strength and enzymes [6,7,8,9,10,11,12,13,14]. Among hundreds of polymers, poly(*N*-isopropylacrylamide) (PNIPAM) and its derivatives have widely been applied in protein science [15,16,17,18]. Schachschal *et al.* [19] reported that the activity of laccase was improved by immobilization in PNIPAM microgel. The stability of horseradish peroxidase (HRP) was improved upon incorporation in PNIPAM-based hydrogels [17]. Generally speaking, these proteins interacted with the polymeric materials through hydrophobic interactions, electrostatic interactions and hydrogen bonds [20].

Despite the abundant literature on protein-polymer interactions, a large portion of the literature is confined to the study of the behavior of immobilized proteins [17,19,21,22,23]. Such observations often lead to an intuitive conclusion that the interactions of polymers with proteins are either very weak or almost zero [24]. Moreover, the behavior of proteins on these surfaces is significantly governed by the surface properties of the polymer and thereby, study of the behavior of proteins on polymeric surfaces does not truly reflect the behavior of a protein in solution. In this regard, the study of the behavior of proteins in solution in the presence of polymers is desirable and important to extend the applications of polymers. Therefore, we aim in this study to explore the behavior of a model protein, bovine serum albumin (BSA), in the presence of thermoresponsive PNIPAM-based polymers in aqueous medium with the aid of biophysical techniques and simulation studies.

BSA is the most abundant globular protein in the plasma. This protein is commonly used as a model protein due to its medicinal importance, low cost, ready availability, stability, water solubility and structural similarity with human serum albumin (HSA) [25,26]. Various endogenous and exogenous ligands are transported by BSA [27]. Structurally, BSA is a single chain of 582 amino acids, non-glycoprotein, cross-linked with 17 cysteine residues [28]. Three types of intrinsic fluorophores are present in BSA: tryptophan (Trp), tyrosine (Tyr) and phenylalanine (Phe) [29]. There are two tryptophan residues in BSA: Trp-212 is located in a hydrophobic binding pocket, and Trp-134 on the surface of molecule [30]. The BSA molecule is made up of three homologous domains (I, II, III) that can be divided into nine loops (L1–L9) by 17 disulphide bonds. Each domain in turn is the product of two subdomains (IA, IB, *etc.*) [31]. Trp-134 is in the first domain, and Trp-212 is in the second domain. All these structural features make BSA one of the best models to understand the physicochemical basis of polymer-protein interactions.

Herein, we prepared copolymers, (poly(*N*-isopropylacrylamide-co-ethylene glycol methacrylate-*co*-acrylic acid)-1 (P(NIPAM-*co*-PEGMA-AA)-1; abbreviated as PGA-1) and poly(*N*-isopropylacrylamide-*co*-ethylene glycol methacrylate-co-acrylic acid)-1.5 (P(NIPAM-*co*-PEGMA-AA)-1.5; abbreviated as PGA-1.5)) by keeping the mole ratio of *N*-isopropyl acrylamide (NIPAM) unchanged, while changing the mole ratios of polyethyleneglycol methacrylate (PEGMA) and acrylic acid (AA). Polymers based on these components have been explored for a wide range of applications in multiple fields. For instance, poly(*N*-isopropylacrylamide) (PNIPAM) is a well-known environmentally sensitive polymer, which shows lower critical solution temperature (LCST) behavior in water [32]. This characteristic behavior of PNIPAM aqueous systems has been studied for use in a number of biomedical fields, such as drug delivery systems [33,34], tissue engineering [35], cell therapy [36] and so on. Being water soluble, nontoxic and non-immunogenic in nature, PEGMA is frequently used for introduction into a polymer structure. Such introduction offers several biological advantages including a prolonged lifetime in the bloodstream due to decreased uptake of PEG-conjugates by the reticuloendothelial system of the body, thereby reducing the toxicity and improving the biocompatibility of the PEG-containing polymer [37]. Further, it was shown that poly(acrylic acid)-based drug carriers were successfully applied in delivering bioadhesive drugs [38]. All these excellent applications inspired us to prepare copolymers consisting of NIPAM, PEGMA and AA. Therefore, we believe that the copolymers based on these monomers are suitable model systems to understand the behavior of biomolecules in the presence of polymers. Further, the present study also presents the behavior of BSA in response to changes in the chemical composition of these responsive polymers. Molecular docking simulation studies provided information about the type and strength of the copolymer-protein interactions. Since the study of the behavior of BSA in the presence of responsive polymers plays a key role in determination of the polymer’s biocompatibility for various biomedical applications and thus helps in the rational design of effective therapeutic carriers, the present results may pave the way to construct PNIPAM-based biomedical devices.

## 2. Experimental Section

### 2.1. Materials

The monomers *N*-isopropylacrylamide (NIPAM), and acrylic acid (AA) were provided by Acros (Fairlawn, NJ, USA). *N*,*N*,*N*′,*N*′-tetramethylethylenediamine (TEMED), ammonium persulfate (APS), *N*,*N*′-methylenebisacrylamide (MBAA) (cross-linker), and poly ethylene glycol methacrylate (PEGMA, *M*_n_ = 360) were obtained from the Aldrich Chemical Co. (St. Louis, MO, USA). BSA was obtained from Sigma-Aldrich (St. Louis, MO, USA). Deionized (DI) water with a resistivity of 18.2 MΩ·cm was obtained from Roda purification system (Te Chen Shen Engineering Co., Ltd., Taichung, Taiwan) and used for all sample preparation. All the chemicals were used as received. All the prepared hydrogel samples were lyophilized using a freeze-dryer before the measurements.

### 2.2. Synthesis of P(NIPAM-co-PEGMA-co-AA) Copolymers

The procedure for the synthesis of these copolymers can be found elsewhere [39]. P(NIPAM-*co*-PEGMA-*co*-AA) copolymers were prepared by following the procedure described here. Initially, PEGMA was mixed with AA thoroughly. Subsequently, the resultant solution was transferred into a three-necked round-bottom flask containing NIPAM and MBAA in phosphate buffered saline. The reaction mixture was subjected to stirring for 30 min with continuous N_2_ gas purging. Finally, to this solution, TEMED and APS were added and reacted for 19 h at room temperature under nitrogen atmosphere. The feed ratio of [NIPAM]/[PEGMA]/[AA]/[APS]/[MBAA] in P(NIPAM-*co*-PEGMA-*co*-AA)-1 is 9.54 × 10^−5^/1.87 × 10^−5^/1.87 × 10^−5^/3.94 × 10^−7^/2.36 × 10^−7^ mol·L^−1^, respectively, and the feed ratio of [NIPAM]/[PEGMA]/[AA]/[APS]/[MBAA] in P(NIPAM-co-PEGMA-co-AA)-1.5 is 9.54 × 10^−5^/2.25 × 10^−5^/1.5 × 10^−5^/3.94 × 10^−7^/2.36 × 10^−7^ mol·L^−1^, respectively. The codes 1 and 1.5 indicate the mole ratios of PEGMA to AA in the copolymer. All the hydrogels were freeze-dried before use. The results for characterization and phase transition of these polymers can be found in our recent work [39]. In our previous work, these polymers were abbreviated as PGA-4 and PGA-7 based on the weight fractions of PEGMA and AA. However, in the present study, the names have been changed from PGA-4 and PGA-7 to PGA-1 and PGA-1.5, respectively, for the sake of convenience.

### 2.3. Sample Preparation

Aqueous solutions of the copolymers at various concentrations (0.05, 0.1 and 0.5% *wt*/*vol*) were prepared for all the measurements by following the procedure, with minimal modifications, reported in previous literature [40]. First, an appropriate amount of freeze-dried copolymer was mechanically ground. This mechanically ground polymer sample was dissolved in a sufficient amount of DI water to obtain the required polymer concentrations (0.05, 0.1 and 0.5% *wt*/*vol*). The solution was rigorously stirred for four days. After stirring, the samples were additionally sonicated for 30 min. Finally, to this solution, the required amount (0.5 mg/mL) of BSA was added.

### 2.4. Fluorescence Intensity Measurements

Fluorescence intensities of the samples were obtained using a Hitachi F-7000 fluorescence spectrophotometer (Tokyo, Japan) with xenon lamp (150 W) as the light source. All the measurements were done at room temperature and atmospheric pressure. The emission spectra were recorded with a slit width of 2.5/2.5 nm and a PMT voltage of 720 V. The scan speed was 1200 nm·min^−1^. Tryptophan (Trp) of BSA was selected as an intrinsic fluorescent probe and excited at the wavelength (λ_exc_) of ~285 nm for the emission spectra.

### 2.5. Circular Dichroism (CD) Spectroscopy

Circular dichroism spectroscopy (Jasco J-715 spectropolarimeter, Tokyo, Japan) was used to record the structural changes of BSA in aqueous solution induced by the addition of copolymers. The CD spectrum was measured in the wavelength ranging from 190 to 250 nm, with a 0.2 nm step resolution, a 50 nm/min speed, a 2 s response time, and a 1 nm bandwidth. Each sample spectrum was obtained by averaging six spectra.

### 2.6. Differential Scanning Calorimetry (DSC)

All the calorimetric measurements were performed on a nano DSC III (TA instruments, Wood Dale, IL, USA) and the data were used to measure the melting temperature (*T*_m_) of BSA in the absence and presence of polymers. Before performing the experiment, the instrument was calibrated with pure water. A certain amount of bubble-free solution was placed into a sample cell, while the reference cell was filled with an appropriate blank sample of the same solvent media without BSA, then capped, and sealed using a press. The cells were allowed to stabilize at 20 °C and then heated to 75 °C with a heating rate of 2.0 °C/min. Enthalpy change (Δ*H*_cal_) and the *T*_m_ of the sample were determined with the aid of software, which accompanied the instrument.

### 2.7. Molecular Docking (MD) Simulations

The molecular docking simulations to understand the interactions between the BSA (PDB No.: 4F5S) and copolymer was performed using the CLC Drug Discovery Workbench (trail version-2.5, Aarhus, Denmark) downloaded from CLC Bio website [41], with the default parameter settings [42]. Energy minimization for the BSA structure was performed using the YASARA SERVER [43]. This server performs an energy minimization using the YASARA force field [44]. The structure of the copolymer was created by using an equal number of monomers units, with the help of Chem Biodraw Ultra. The geometry of the polymer was optimized using Chem Biodraw Ultra with the default settings and the structure was saved in MDL Molfile (* mol) format for further docking studies. The copolymer was transformed to a ligand before performing the docking studies. According to the developers, the docking score used in the Drug Discovery Workbench is the PLANTS_PLP_ score [45]. The score has a good balance between accuracy and evaluation time. The score mimics the potential energy change when the protein and ligand come together. The structural visualization for the interactions between BSA and the copolymer was performed using the CLC Drug Discovery Workbench.

## 3. Results and Discussion

**Figure 1:**
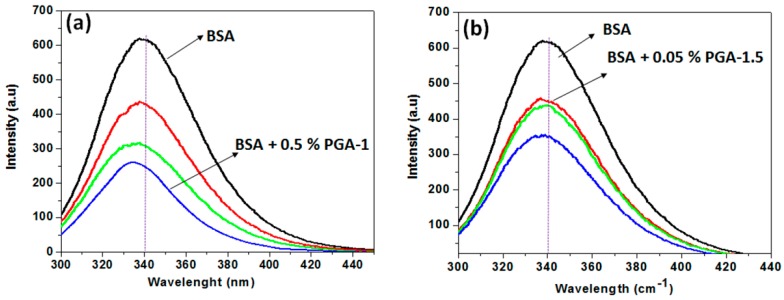
Fluorescence intensities of BSA in the absence and presence of PGA-1 (**a**); and PGA-1.5 (**b**) at various concentrations. The concentrations of polymers are 0.05% (red color line), 0.1% (green color line), and 0.5% (blue color line). The excitation wavelength of Trp is 295 nm.

In order to gain more insight into the behavior of BSA in the presence of P(NIPAM-*co*-PEGMA-*co*-AA) copolymers, the influence of the polymers on the fluorescence spectrum of the protein was investigated (Figure 1). The fluorescence measurements provide the information regarding the molecular environment of the chromophore. BSA contains intrinsic fluorescence fluorophores, tryptophan (Trp), and 18 tyrosine residues that are responsible for its fluorescence emission. Trp is located in subdomain IIA within a hydrophobic pocket, whereas tyrosines are distributed along the whole polypeptide chain. These fluorophores can be excited at the wavelength (λ_exc_) of ~285 nm; however most of the fluorescence attributed to Trp because of the efficient resonance energy transfer (RET) from tyrosine to tryptophan [46]. Since the Trp fluorophore can be excited at the wavelength (λ_exc_) of ~285 nm, the excitation of all other amino acids in the protein can be avoided as these absorb at shorter wavelength [20]. The fluorescence of Trp in the protein is sensitive to environmental changes such as polarizability [47], and thus it can be used as an optical probe to analyze the effect of co-solute on the conformational changes of the protein [48]. The changes in the fluorescence parameters such as maximum emission wavelength (λ_emi_) and intensity (*I*_max_) can be related to the conformational changes of the protein [49].

Figure 1 shows the fluorescence spectra of BSA in the absence and presence of copolymers (PGA-1 & PGA-1.5) in water, at atmospheric temperature, with different concentrations of the copolymers. As shown in Figure 1, the maximum fluorescence intensity (*I*_max_) (619.5 a.u) of BSA was observed at ~341 nm in the absence of the polymers, characteristic of Trp, indicating that this residue is relatively buried inside the BSA [50]. The addition of both the polymers resulted in substantial decrease of *I*_max_ of BSA. However, the effect of PGA-1.5 (Figure 1b) on the intrinsic fluorescence intensity of BSA was less pronounced as compared to that in the presence of PGA-1 (Figure 1a). These results suggest that the ability of PGA-1 to unfold BSA was stronger, and thus this polymer strongly quenches the fluorescence of BSA. It is reported that the folded conformation of BSA in water is attributed to the formation of a water shell around the BSA [49]. In the present study, the equal distribution of monomers, PEGMA and AA, cause PGA-1 to be more hydrophilic [39], and thereby this polymer can easily alter the hydration shell around the BSA. Moreover, the optimum amount of PEGMA in PGA-1 can increase the bulkiness of the backbone of polymer, and thereby, restricts the flexibility of the entire copolymer system and results in more hydrophilicity by hindering the formation of the intramolecular hydrogen bonds [39,51]. Therefore, the behavior (flexible/stiff) of the polymer is the result of intermolecular hydrogen bonds. Moreover, strong intramolecular hydrogen bonding at certain composition makes the polymer less hydrophilic, despite the higher content of the hydrophilic monomer [51]. Therefore, it is very important to fabricate the polymer with optimal chemical composition.

**Scheme 1:**
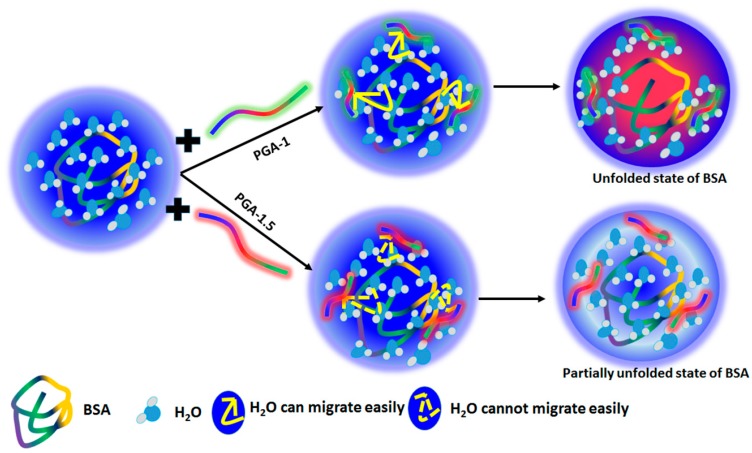
Schematic demonstration of the unfolding mechanism of BSA in the presence of PGA-1 and PGA-1.5.

For a more intuitive understanding of the unfolding mechanism of BSA in the presence of copolymers (represented by the three color segments (blue, red and green, and these three colors represent three monomer units in the copolymers), a schematic illustration is presented in Scheme 1. As illustrated in the scheme, BSA is fully hydrated in the absence of the polymers. However, the addition of PGA-1 (represented by the green line in Scheme 1) to the protein aqueous solution results in competition for water molecules between the polymer and protein. Due to the higher hydrophilic nature of PGA-1, the water molecules from the surface of the protein can migrate easily to this polymer and thus make BSA unfold easily (represented by the red area in Scheme 1). On the other hand, despite the unfolding of BSA in the presence of PGA-1.5 (represented by the red line in Scheme 1), the extent of unfolding seems to be smaller when compared that in the presence of PGA-1. Due to the higher hydrophobic nature, PGA-1.5 cannot attract water molecules easily from the hydration shell around the protein and lead to partial unfolding (represented by light blue area in Scheme 1). Therefore, the higher fluorescence intensity of BSA was observed in the presence of PGA-1.5 (Figure 1b). The enzyme activity mainly depends on the volume of the hydration shell around the protein, but not on the volume of bulk water [52]. A similar decrease in the *I*_max_ of Trp was observed when exposed to the water molecules in the presence of additives [53,54]. These results indicate that the conformation of BSA was significantly altered by the addition of both polymers. Moreover, the *I*_max_ of BSA decreased with an increase in the concentration of both the copolymers PGA-1 and PGA-1.5 (Figure 1a,b). For instance, the *I*_max_ of BSA was 451 a.u in the presence of PGA-1.5 at a concentration of 0.05% (*w*/*v*), while *I*_max_ was found to be 356 a.u at a concentration of 0.5% (*w*/*v*) (Figure 1b). On the other hand, the *I*_max_ of BSA was 435 a.u in the presence of PGA-1 at a concentration of 0.05% (*w*/*v*), while *I*_max_ was found to be 264 a.u at a concentration of 0.5% (*w*/*v*). The decrease in *I*_max_ by increasing the concentration of the polymer can be regarded to the maximum change in the conformation of the protein. It is worth mentioning here that the 0.05% (*w*/*v*) PGA-1.5 was able to quench the fluorescence intensity by altering the hydration layer around the protein. However, the fluorescence intensity was not further quenched significantly with an increase in the concentration of PGA-1.5 from 0.05% (*I*_max_ = 451 a.u) to 0.1% (*w*/*v*) (*I*_max_ = 437 a.u) (Figure 1b). This can be attributed to the higher hydrophobic nature of PGA-1.5. Being more hydrophobic, PGA-1.5 cannot attract the water molecule in the close vicinity of the protein surface at a concentration of 0.1% (*w*/*v*). However, the water molecules from the close vicinity of protein surface can migrate by the addition of a higher amount of polymer as evidenced from the quenching of fluorescence by the addition of 0.5% (*w*/*v*) PGA-1.5 (Figure 1b). A similar type of decrease was observed in the *I*_max_ of serum protein with the addition of the copolymers [37].

Further, Figure 1a indicates a shift in the λ_emi_ of BSA towards a shorter wavelength (hypsochromic shift) with the addition of the polymers. For instance, in the absence of copolymers, BSA has λ_emi_ at ~340 nm. However, with the addition of PGA-1, the λ_emi_ shifted to lower wavelengths (338, 337 and 334 for 0.05, 0.1 and 0.5 % (*w*/*v*), respectively). On the other hand, despite the blue shift in λ_emi_ of BSA by the addition of PGA-1.5, the extent of the shift was less significant than with PGA-1. For instance, the λ_emi_ shifted to ~337 nm by the addition of 0.5% (*w*/*v*) PGA-1.5. This blue shift in λ_emi_ indicated that the tryptophan residue experienced a more hydrophobic (less polar) environment in the presence of the polymers [55]. Azegami *et al.* [55] reported a hypsochromic shift of λ_emi_ of serum protein upon interaction with high molecular weight PEG. They explained this hypsochromic shift of λ_emi_ on the basis of protein-protein complex formation induced by large PEG chains. The hypsochromic shift in the λ_emi_ of BSA observed upon the complexation with the copolymers can be explained on the basis of a similar phenomenon where the PEGMA segments in the copolymers induced a protein-protein interaction. These BSA molecules may interact with themselves or with nearby PEGMA chains present in the copolymer, which can result in the hypsochromic shift of emission maximum as shown in Figure 1. The presence of PEGMA in the copolymers at higher content resulted in higher hydrophobicity with respect to the PGA-1.5 and thus, this polymer is not that effective in perturbing the hydration layer as evidenced from Figure 1b. These results are consistent with the results of existing studies [37]. Further, Zhao *et al.* [56] reported that the fluorescence intensity of tryptophan buried inside BSA is much stronger when compared to that exposed to the water molecules in aqueous solution at the same concentration.

CD spectroscopy has been proved as a technique of choice to extract information regarding the secondary structure of proteins and nucleic acids [57]. BSA exhibits two characteristic negative bands in the UV region at 208 and 220 nm, indicating an α-helical structure of the protein [53]. The negative band at 208 nm is due to the exciton splitting of the lowest peptide π→π* transition, while the negative band at 220 nm is due to the peptide n→π* transition [58].

**Figure 2:**
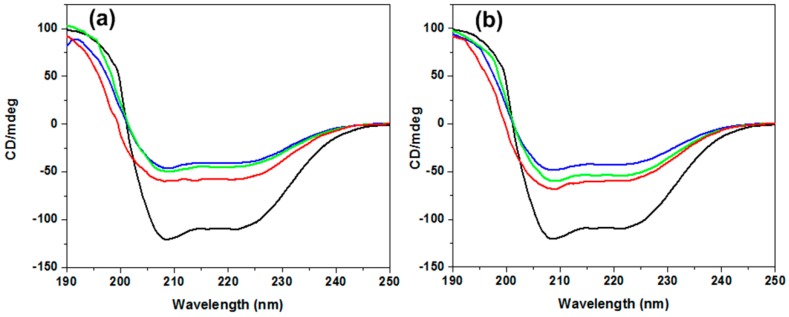
Far-UV CD spectra of BSA (black line) and BSA in the presence of PGA-1 (**a**); and PGA-1.5 (**b**). The concentrations of polymers are 0.05% (red line), 0.1% (green line), and 0.5% (blue line).

Typical far-UV CD spectra of BSA in the absence and presence of PGA-1 and PGA-1.5 at different concentrations are presented in Figure 2. BSA showed a typical CD signature with a high alpha helical content with considerable negative ellipticity at ~208 and ~220 nm in the far UV range in the absence of copolymers as shown in Figure 2. Neither of the polymers showed the CD signal in the range of 190–250 nm, and thus the observed CD was solely due to the peptide bonds of the protein. The negative bands at ~208 and ~220 nm were found to be collapsed by the addition of PGA-1 (Figure 2a) and PGA-1.5 (Figure 2b). These results indicated that the intramolecular forces responsible for maintaining the secondary structure were altered in the presence of these polymers. Such changes can be viewed from the alterations in the circular dichroism (CD) spectrum of the protein as presented in Figure 2. With an increase in copolymer concentration, a further reduction in the magnitude of negative ellipticity of the CD bands was observed. However, in the presence of the PGA-1.5 copolymer (Figure 2b), the loss of magnitude of negative ellipticity was smaller when compared to PGA-1 (Figure 2a). This is in agreement with the fluorescence quenching data presented earlier. A reduction in the magnitude of negative ellipticity of the CD bands of BSA occurred to a greater extent in the presence of PGA-1, as was pointed out previously in the quenching experiments, *i.e.*, as PGA-1 was added, it easily altered the hydration shell around BSA, and thus led to BSA undergoing the unfolding process easily. The polymer-induced alterations in the peaks at 208 and 220 nm suggested that the polymers were effective in initiating partial unfolding of the protein chain. Similar partial unfolding behaviors of the protein chains due to the presence of ionic liquids and dendrimers in the aqueous protein solutions have also been reported [59,60].

**Figure 3:**
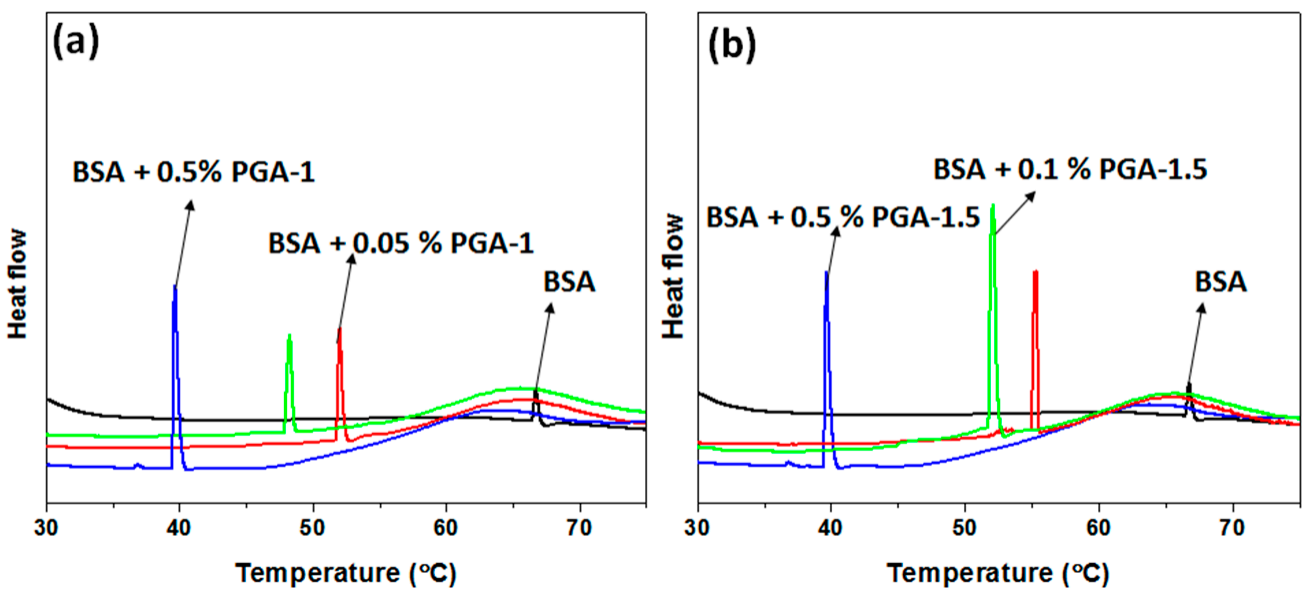
DSC thermograms of BSA in the absence and presence of PGA-1 (**a**) and PGA-1.5 (**b**) at various concentrations. The concentrations of the polymers are 0.05% (red line), 0.1% (green line), and 0.5% (blue line). Tm represents the highest point of relative heat.

The thermal unfolding of the serum albumin protein proceeds in multiple steps; [61] native (N) → extended (E) → unfolded (U). In the extended form (E) (at ≤55 °C), despite the displacement of domains I and II, the protein almost maintains its native conformation, whereas in the unfolded state (U), domain II starts to melt, disrupting the secondary and tertiary structure of the protein. Moreover, the N → E transition is reversible. However, for the E → U transition, the native state cannot be retrieved upon decreasing the temperature. The change in the *T*_m_ values of the BSA upon the addition of the copolymers was obtained from DSC thermograms (Figure 3). The *T*_m_ was determined as the highest point of the heat flow in the DSC thermograms. As shown in Figure 3, BSA has reduced *T*_m_ values in the presence of copolymers. For instance, the *T*_m_ of BSA decreased from 66.7 °C (in the absence of copolymers) to 51.9 °C (0.05% *w*/*v*), 48.1°C (0.05% *w*/*v*) and 39.6 °C (0.05% *w*/*v*) in the presence of PGA-1. This decrease in the *T*_m_ of BSA upon the addition of polymers was attributed to the perturbation of the hydration layer around the BSA surface. However, the extent of the decrease in *T*_m_ was greater in the presence of PGA-1 at a given concentration. For instance, at 0.05% (*w*/*v*) PGA-1 (Figure 3a), *T*_m_ of BSA decreased by 14.8 °C, whereas the *T*_m_ decreased by 11.5 °C in the presence of PGA-1.5 (Figure 3b). This result implied that the thermal stability of BSA decreased, and both copolymers acted as structure destabilizers. However, PGA-1 can be considered as a relatively stronger destabilizer as it shifts *T*_m_ to a greater extent (Figure 3a). The thermal stability of the folded protein is mainly governed by the ordering of water molecules around the hydrophobic groups of the protein [62]. The loss of the hydration layer around the peptide of BSA resulted from the preferential interactions between the polymer and water molecules. Moreover, it is worth mentioning that each sample exhibited the broad peak after the *T*_m_ value in the DSC thermogram as shown in the figure, indicating the aggregation of the BSA-copolymer complex at higher temperatures.

**Figure 4:**
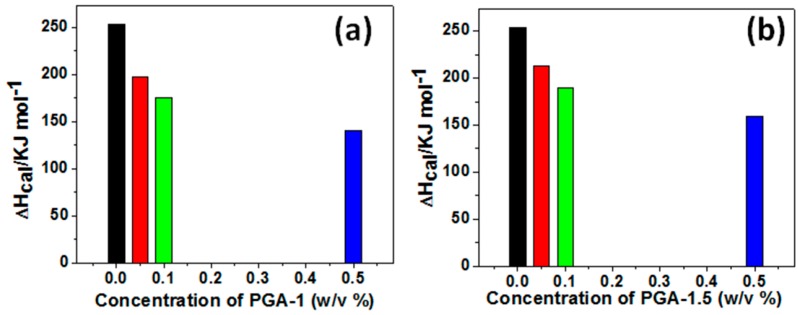
The changes in Δ*H*_cal_ of BSA upon addition of PGA-1 (**a**); and PGA-1.5 (**b**) at various concentrations.

The differences in the behavior of the *T*_m_ of BSA in response to the PGA-1 and PGA-1.5 copolymers at the same concentration can be attributed to the differences in the PEGMA/AA ratios of these copolymers. The contents of PEGMA and AA are the same in the PGA-1 copolymer. Such equal distribution of monomers makes the polymer relatively more hydrophilic and thus PGA-1 can easily perturb the hydration layer around the protein. In contrast to this, with further enhancement of PEGMA content as in PGA-1.5 copolymer, the resulted copolymer becomes relatively less hydrophilic. Due to this relatively less hydrophilic nature, PGA-1.5 needs more energy to perturb the hydration layer around the proteins and thus T_m_ was observed at higher temperatures. The relative hydrophilic natures of these copolymers can be monitored from their lower critical solution temperatures (LCST) (LCST values of these copolymers were measured from the DSC thermograms) as reported in our previous work [32]. These results reveal that the distribution of monomers plays a significant role in governing the hydrophilic/hydrophobic nature of the copolymer, and consequently their effect on the behavior of proteins. Further, there are reports where the distribution of monomers controlled the hydrophilic/hydrophobic properties of the resulted copolymer [63,64]. Further, the unfolding enthalpy (ΔH_cal_) of BSA (253 KJ·mol^−1^) decreased upon addition of PGA-1 (198, 145 and 140 KJ·mol^−1^ for 0.05%, 0.1% and 0.5% (*w*/*v*), respectively) and PGA-1.5 (212, 189 and 160 KJ·mol^−1^ for 0.05%, 0.1% and 0.5% (*w*/*v*), respectively) as shown in Figure 4 and strongly consistent with the *T*_m_ values. Considering the fluorescence, CD and DSC results, we believe that both polymers can change conformation. In order to obtain more insight into the molecular level interactions, we performed molecular docking simulations.

Molecular docking simulations can be used as a complementary tool to understand the protein-ligand interactions at a molecular level. The molecular docking simulation was performed using the CLC Drug Discovery Workbench. The first step in molecular docking is to identify the most probable and potential binding pockets within the protein structure. Under the default settings, CLC identifies a number of binding pockets lying within the range of 20 to 632.83 Å in the structure of BSA. Therefore, we have confined our search for the binding pockets that are greater than 100 Å. Moreover, analysis of several protein-ligand structures from the Protein Data Bank (PDB) revealed that most of the binding sites were the largest pockets found on the protein [65].

**Figure 5:**
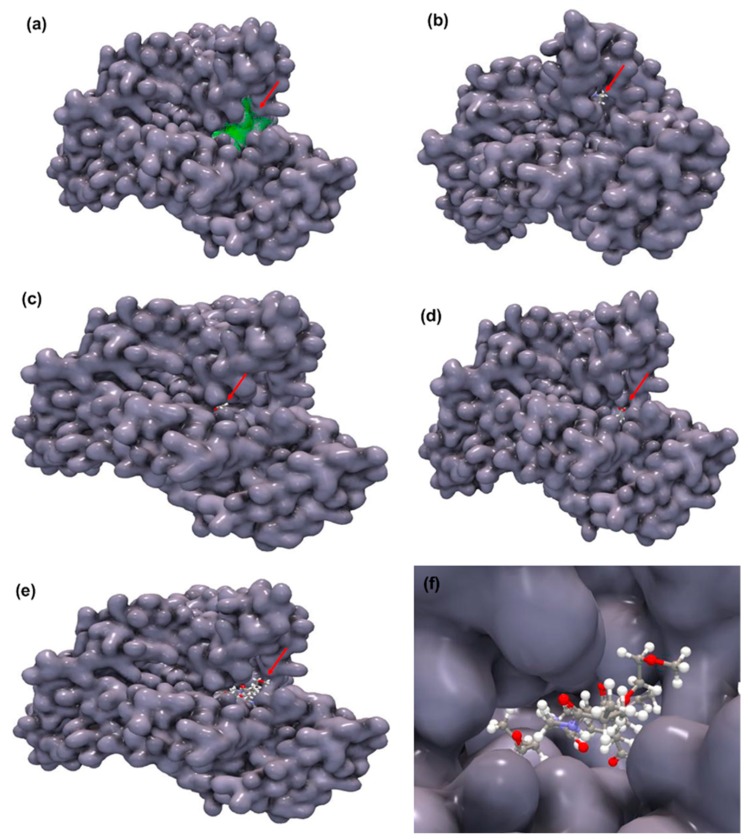
Molecular docking snapshots of the potential binding sites of BSA (**a**); interactions of NIPAM with BSA (**b**); interactions of PEGMA with BSA (**c**); interactions of AA with BSA (**d**); interactions of copolymer with BSA (**e**); and the magnified images for interactions of the copolymer with the largest binding pocket of the BSA (**f**). The red arrow indicates the position of the monomer or copolymer in the protein.

As presented in Figure 5a (the green areas), three potential binding sites were identified over the surface of the BSA with the molecular dimension of 632.83, 422.40 and 127.49 Å. On the other hand, in order to compare the magnitude of interaction of the copolymer with the BSA, we have individually docked the monomers of the copolymer, such as NIPAM, AA, and PEGMA, with the BSA, and the H-bond score is presented in Table 1. Apparently, if arranged in the order of increasing hydrogen bonding score, the order is NIPAM < PEGMA < acrylic acid < copolymer. The docking result shows that NIPAM interacts only with the Tyr 137 residue (Figure 5b), whereas, PEGMA interacts with the Leu 189 and Set 192 residues (Figure 5c). On the other hand, AA shows hydrogen bonding with Arg 144, Leu 112 and Asp 111 residues (Figure 5d). The most promising result is with the copolymer that interacts with Ser 109, Arg 144, Arg 185, Glu 424, Arg 427 and Ser 428 amino acid residues within the binding pocket (Figure 5e). The possible interacting model for the copolymer involved in the interaction is depicted in Figure 5f, for the largest binding pocket of 632.83 Å.

The difference in the hydrogen bonding for the NIPAM, PEGMA, AA and the copolymer can also be accounted for the molecular structure. It is a well-known fact that the docking process follows the lock and key mechanism and thus, if the properties (such as size, shape and charge, *etc.*,) of the ligands match with the properties of binding pocket a, high docking score can be achieved. A similar phenomenon was observed in the case of NIPAM, PEGMA, AA and the copolymer. The size of the copolymer is obviously higher as compared to the rest of its components. This indicates that the copolymer fits much better into the binding pockets of BSA during docking and hence, we observe the highest docking score for the copolymer in all of the three predicted binding pockets of BSA. Due to the perfect match in the size of the copolymer with the binding pocket, the surrounding amino acids come close enough to form strong hydrogen bonding with the polymer. Hence, due to the preferable interaction of the copolymer with the protein surface, the solvation structure of water molecules around the binding pockets are displaced and hence, the structure of the protein is disturbed. Moreover, the intermolecular hydrogen bonding interaction of amino acid residues with the copolymer decreased the hydrophobicity of the system. Therefore, we observed quenching in the fluorescence as well as thermal instability of BSA in the presence of the copolymer.

## 4. Conclusions

The applications of stimuli-responsive polymers as therapeutic carriers can be amplified by the comprehensive understanding of their influences on behaviors of biomolecules. In this context, the stability of the model protein, BSA, in the presence of PNIPAM based thermoresponsive copolymers with different PEGMA/AA ratios was evaluated in aqueous solution with the aid of experimental and docking studies. Our experimental and docking results showed that the chemical structure and composition of the constituted monomers of the polymers play a significant role in governing the behavior of the protein in aqueous solution. Molecular docking simulation revealed that the surrounding amino acids come close enough to form strong hydrogen bonding with the polymer because of the perfect match in the size of the copolymer with the binding pocket. Hence, due to the preferable interaction of the copolymer with the protein surface, the solvation structure of water molecules around the binding pockets is displaced and hence, the structure of the protein is perturbed. The thermal stability and secondary structure of BSA are greatly affected by PGA-1. The higher destabilizing ability of PGA-1 can be attributed to the relative higher hydrophilicity. PEGMA and AA are distributed equally in the PGA-1 copolymer. Such equal distribution of monomers makes the polymer relatively more hydrophilic and thus PGA-1 can easily perturb the hydration layer around the protein. The present results can pave the way for the correct selection of chemical structure and composition of monomers to prepare the stimuli responsive polymers that can be used for biomedical applications.

## Figures and Tables

**Table 1:** Docking score and hydrogen bond (H-bond) score for the interaction of the copolymer, PEGMA, NIPAM and AA with the best selected docking position of BSA.

Molecular dimensions of the binding sites	Docking score	H–bond score
Copolymer		
632.83 Å	−61.86	−19.13
422.83 Å	−45.87	−14.57
127.49 Å	−59.78	−4.00
PEGMA		
632.83 Å	−27.44	−4.00
422.83 Å	−27.48	−8.00
127.49 Å	−31.39	−3.98
NIPAM		
632.83 Å	−23.99	−0.72
422.83 Å	−27.68	−4.00
127.49 Å	−28.78	0.00
AA		
632.83 Å	−22.91	−9.72
422.83 Å	−21.73	−11.62
127.49 Å	−20.50	−4.00
